# Factors associated with perinatal mortality in Nepal: evidence from Nepal demographic and health survey 2001–2016

**DOI:** 10.1186/s12884-019-2234-6

**Published:** 2019-03-11

**Authors:** Pramesh Raj Ghimire, Kingsley E. Agho, Andre M. N. Renzaho, Monjura K. Nisha, Michael Dibley, Camille Raynes-Greenow

**Affiliations:** 1 School of Science and Health, Western Sydney University, Locked Bag1797, Penrith, NSW 2571 Australia; 2 School of Social Sciences and Psychology, Western Sydney University, Locked Bag1797, Penrith, NSW 2571 Australia; 3 Sydney School of Public Health, University of Sydney, Edward Ford Building (A27), Sydney, NSW 2006 Australia

**Keywords:** Perinatal mortality, Extended perinatal mortality, NDHS, Nepal

## Abstract

**Background:**

Perinatal mortality is a devastating pregnancy outcome affecting millions of families in many low and middle-income countries including Nepal. This paper examined the more distant factors associated with perinatal mortality in Nepal.

**Methods:**

A sample of 23,335 pregnancies > 28 weeks’ gestation from the Nepal Demographic and Health Survey datasets for the period (2001–2016) was analysed. Perinatal Mortality (PM) is defined as the sum of stillbirth (fetal deaths in pregnancies > 28 weeks’ gestation) and early neonatal mortality (deaths within the first week of life), while Extended Perinatal Mortality (EPM) is denoted as the sum of stillbirth and neonatal mortality (deaths within the first 28 days of life). Rates of PM and EPM were calculated. Logistic regression generalized linear latent and mixed models (GLLAMM) that adjusted for clustering and sampling weight was used to examine the factor associated with perinatal mortality.

**Results:**

Over the study period, the PMR was 42 [95% Confidence Interval (CI): 39, 44] per 1000 births for the five-year before each survey; while corresponding EPMR was 49 (95% CI, 46, 51) per 1000 births. Multivariable analyses revealed that women residing in the mountains, who did not use contraceptives, women aged 15–18 years or 19–24 years, and women having no education were associated with increased PM and EPM. The study also identified households using biomass as cooking fuel, and households who reported unimproved sanitation or open defecation were significantly more likely to experience PM and EPM.

**Conclusions:**

Interventions aimed to improve use of contraceptives, and reduce biomass as a source of cooking fuel are needed to achieve the recommended target of < 12 perinatal deaths per 1000 births by 2030.

**Electronic supplementary material:**

The online version of this article (10.1186/s12884-019-2234-6) contains supplementary material, which is available to authorized users.

## Background

Availability and quality of healthcare of both mother and newborn is reflected in the perinatal mortality rate, and perinatal mortality remains one of the devastating pregnancy outcomes for millions of families in low-and-middle-income countries including Nepal [1]. A recent Lancet systematic review for the Global Burden of Disease estimated that every year over 4 million perinatal deaths occur worldwide; and almost all (98%) of these deaths occur in low-and-middle-income countries, mostly in sub-Saharan Africa and South Asia, including Nepal [2]. The perinatal mortality rate is five times higher in low as compared to high income countries, with 10 deaths per 1000 total births in high income countries; 50 per 1000 total deaths in low and middle income countries and over 60 per 1000 in the most deprived countries [1].

In Asia, the perinatal mortality rate is estimated to be 50 per 1000 births, but as high as 65 per 1000 in South-central Asia, the third-highest rate among the sub-regions [1]. Past studies indicated that preterm birth, intrapartum complications and infections are the three leading causes of perinatal death [3], and millions of these deaths can be averted with high coverage quality interventions along with a population-specific action plan for women who are socially marginalized based on issues such as ethnicity, geography and socioeconomic status [4]. A case-control study conducted in Pakistan identified that maternal literacy, poor socio-economic household, primigravida, and lack of knowledge of family planning were associated with perinatal mortality [5]. Similarly, a population-based cross-sectional study conducted in Bangladesh that examined the impact of maternal excessive body weight on a range of maternal and child health outcomes reported that overweight and obesity among women aged 25 years and older increased the risk of perinatal mortality by 1.8 [95% Confidence Interval (1.5, 2.1)] [6].

Perinatal mortality rates play an increasingly important role in childhood mortality, and there are currently no effective community-based intervention programs in Nepal particularly targeting perinatal mortality including stillbirth. Despite the high burden of perinatal mortality globally, there have been very limited epidemiological studies that examine potential factors associated with perinatal mortality in Nepal. Previous studies conducted in Eastern and Central districts of Nepal found that birth asphyxia, infection, and prematurity were the major causes of stillbirth and neonatal mortality [7, 8]. A multi-centre prospective study conducted in the Jhapa and Kathmandu districts revealed that higher parity (> 4), low birthweight (< 1999 g) and older maternal age (≥35 years) were reported to be associated with PM [9]. In contrast, a cross-sectional study conducted in central Nepal found that teenage women were more likely to report higher perinatal mortality [10]. Similarly, another prospective study conducted in Kathmandu concluded that perinatal mortality occurred more frequently among primigravid women, and those having preterm birth reported a higher risk of perinatal mortality [11]. However, the external validity of these studies was limited because they were not population-based, hence may not be generalised to inform effective interventional policy that will significantly reduce perinatal death. The Nepal Demographic and Health Survey (NDHS) dataset provides an opportunity to examine factors associated with perinatal mortality using a population-based sample. Using a national population-based sample will allow the formulation of an integrated policy and programmatic response to address perinatal mortality in Nepal.

The main aim of this study was to determine more distant factors associated with Perinatal Mortality (PM) and Extended Perinatal Mortality (EPM) by using the four most recent nationally representative household data of NDHS for the years 2001, 2006, 2011, and 2016.

## Methods

### Data sources and sample

This study combined data of NDHS for the years 2001 [12], 2006 [13], 2011 [14] and 2016 [15]; which were nationally representative household surveys, using multistage cluster sampling designs, stratified by geographical regions and urban and rural areas. All four surveys sampling methods were similar and routinely collected with the objective of estimating socio-demographic; and maternal and child health indicators at national and district level.

A total of 32,193 women aged 15–49 years were interviewed in the four surveys (8726 women in 2001 NDHS, 10,793 women in 2006 NDHS, 12,674 women in 2011 NDHS, and 12,862 in 2016 NDHS), with the average response rate over 97%. In the 2001 NDHS [12] women were asked to report all pregnancies they had in their lifetime records of pregnancy loss and the duration of such pregnancies included; whereas in the 2006 NDHS [13], the 2011 NDHS [14], and the 2016 NDHS [15] women were asked to report on any pregnancy loss and the duration of such pregnancy that occurred five years preceding the surveys. Information such as pregnancy, pregnancy loss and duration of pregnancy was used to identify the number of livebirth, stillbirth and pregnancies > 28 weeks’ gestation. For this study 23,335 pregnancies > 28 weeks’ gestation were identified (*N* = 7134 in 2001, *N* = 5671 in 2006, *N* = 5444 in 2011, and *N* = 5086 in 2016). Details of the survey methodology, sampling procedures, and questionnaires are provided in the respective NDHS reports [12–15].

### Outcome

Outcome variables for this study were: (I) Perinatal Mortality (PM) defined as the sum of stillbirth (fetal deaths in pregnancies > 28 weeks’ gestation) and early neonatal mortality (deaths within the first week of life) [12–15], and (II) Extended Perinatal Mortality (EPM) defined as the sum of stillbirth and neonatal mortality (deaths within the first 28 days of life) [12–16].

### Potential explanatory variables

**Fig. 1:**
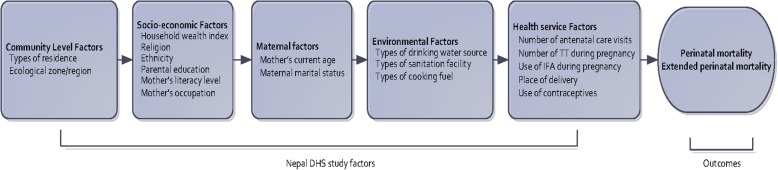
Framework for factors associated with perinatal mortality in Nepal, adopted from Mosley and Chen analytical framework for the study of child survival in low income countries

The selection of potential explanatory variables for this study was based on past studies that have examined factors associated with perinatal mortality in different low-and middle-income countries [9, 17–19]. We also adopted Mosley and Chen’s analytical framework for the study of child survival in low income countries [20]. Figure 1 illustrates the factors used to examine their relation with PM and EPM in that framework. Based on Mosley and Chen’s framework, 19 explanatory variables were classified into five categories as community level factors (types of residence and ecological zone); socioeconomic factors (wealth index, religion, mother’s education, mother’s literacy level, father’s education and mother’s occupation); maternal factors (mother’s current age, maternal marital status, and birth order and birth interval); environmental factors (types of drinking water sources, types of sanitation facility and types of cooking fuel); and health service factors (number of antenatal care visits, number of Tetanus Toxoid (TT) vaccines during pregnancy, place of delivery and use of contraceptives). Birth order and birth interval were combined because a previous study found that the impact of birth order may be mediated by birth interval [21]. A household wealth index variable was constructed using principle component analysis [22] of the common household facilities across four NDHS, 2001–2016 (electricity, radio, television, bicycle, telephone, and main material of floor). For the purpose of this study, the household wealth index was divided into three categories. The bottom 40% of households were arbitrarily referred to as poor households, the next 40% was classified as the middle households and the top 20% was classified as rich households, consistent with previous studies [18, 21].

The World Health Organization and the United Nations Children’s Fund Joint Monitoring Program guidelines were used to categorize the source of drinking water and types of sanitation facility [23]. Categories of source of drinking water included (1) piped water on premises, (2) other improved drinking water sources, (3) unimproved drinking water sources, and (4) surface drinking water sources. Households with piped water system into dwelling were categorised as piped water on premises. Households relying on neighbour’s tap or tubewell, borehole in yard, stone tap, protected well and rainwater for drinking were categorised as other improved drinking water sources. Households who reported unprotected well in-house, unprotected public or neighbour’s well, unprotected spring, bottled water or water from tanker or truck as sources of drinking water were categorised as unimproved water sources. Households who reported river, stream, pond, lake, dam, canal, or irrigation water for drinking were categorised as surface drinking water sources. Likewise, categories of sanitation facility included (1) improved sanitation facilities, (2) unimproved sanitation facilities, and (3) open defecation. Households having flush toilet, ventilated or improved pit latrine, pit latrine with slab or composting toilet were categorised as improved sanitation facilities. Households who reported traditional pit toilet, pit latrine without slab, bucket toilet were categorised as unimproved sanitation facilities. Households relying on bush or open field for defecation were categorised as open defecation.

### Statistical analysis

Weighted frequencies for all explanatory variables were calculated (Table 2). Perinatal Mortality Rate (PMR) and Extended Perinatal Mortality Rate (EPMR) with 95% confidence interval across all explanatory variables were calculated (Table 2). The sample was restricted to five years preceding each survey. Data were analysed using STATA 14.1 (Stata Corp, College Station, Texas, US). SVY functions were used to adjust for sampling weights. Logistic regression generalized linear latent and mixed models (GLLAMM) with the logit link and binomial family [24] that adjusted for clustering and sampling weights were used to measure the level of association between outcomes and explanatory variables. Multivariate analysis was conducted using staged technique described by Victora et al. [25] and based on this technique, the effect of distal determinants can be assessed without adjustment of proximal or intermediate determinants [21]. Therefore, at the first stage, all community-level factors (more distal determinants) were assessed in the baseline multivariable model with manual backward elimination to keep statistically significant factors (model 1). Second, socioeconomic factors were added with model 1 and manual backward elimination process was repeated to keep statistically significant factors (model 2). This procedure was followed when maternal, environmental and health service variables were included in the third (model 3), in the fourth (model 4) and in the fifth stage (model 5) respectively. Variables significantly associated at the 5% significance level were included in model 5 and reported in the study. In the final model (Model 5), we tested and reported any collinearity. Unadjusted and adjusted odd ratios with 95% confidence interval of the final model were reported.

“We double checked our findings by re-normalising the sampling weights for each year of survey to add up to 1. This process involved computing the total sum of weights for each survey round and divide each year of survey sampling weights with the total sum of weights. The results obtained from using each year of survey sampling weights presented in the manuscript and those obtained from re-normalized sampling weights were similar (see Additional files 1 and 2).

## Results

**Fig. 2:**
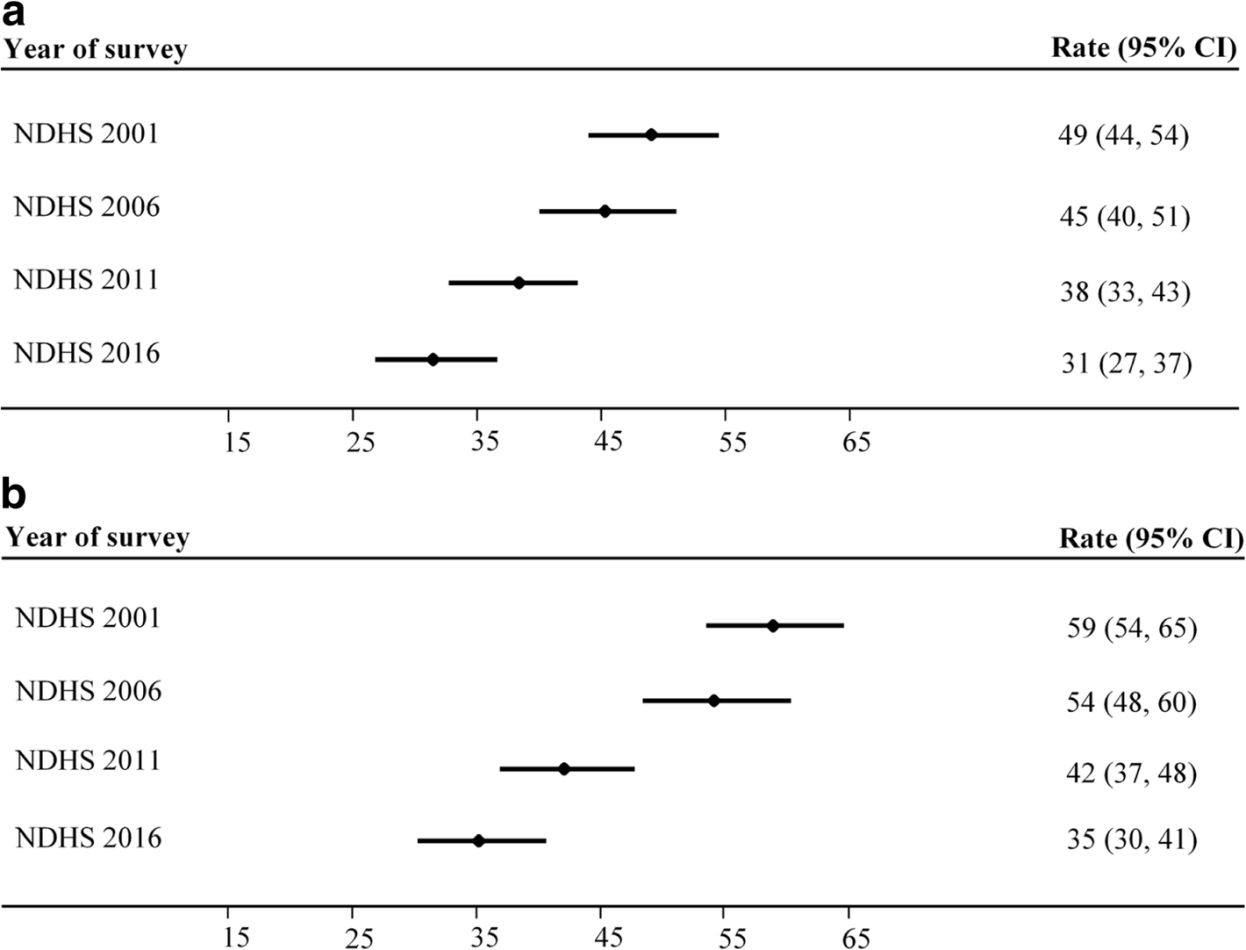
**a** Rate and 95% Confidence Interval (CI) of perinatal mortality per 1000 births in NDHS 2001, 2006, 2011, and 2016. **b** Rate and 95% Confidence Interval (CI) of extended perinatal mortality per 1000 births in NDHS 2001, 2006, 2011, and 2016

Over the study period (2001–2016), PMR was 42 (95% CI: 39, 44) per 1000 births; whereas EPMR was 49 (95% CI: 46, 51) per 1000 births (Table 1). PMR and EPMR decreased significantly in 2011 and 2016 compared to 2001 (Fig. 2a, b). Similarly, PMR and EPMR decreased significantly in 2016 compared to 2006. However, there was no significant decrease in PMR and EPMR in 2016 compared to 2011.

**Table 1:** Rates and 95% Confidence Intervals (CI) of stillbirth, early neonatal mortality, late neonatal mortality, neonatal mortality, perinatal mortality, and extended perinatal mortality in Nepal (2001–2016)

Birth and mortality rate	Birth/Mortality	Rate (95% CI)
All births, N^a^	23,335	–
Stillbirth, rate^b^ per 1000 births (95% CI)	414	18 (16, 19)
Early neonatal mortality, rate^c^per 1000 live births (95% CI)	561	24 (22, 27)
Late neonatal mortality, rate^d^per 1000 live births (95% CI)	160	7 (6, 8)
Neonatal mortality, rate^e^ per 1000 live births (95% CI)	721	31 (29, 34)
Perinatal mortality, rate^f^ (95% CI)	975	42 (39, 44)
Extended perinatal mortality, rate^g^ (95% CI)	1135	49 (46, 51)

^a^N included stillbirths and live births from pregnancies > 28 weeks’ gestation

^b^The rate of stillbirth was calculated from the number of stillbirths divided by all births multiplied by 1000

^c^Early neonatal mortality rate is early neonatal mortality divided by total live births multiplied by 1000

^d^Late neonatal mortality rate is late neonatal mortality divided by total live births multiplied by 1000

^e^Neonatal mortality rate is neonatal mortality divided by total live births multiplied by 1000

^f^Perinatal mortality rate is stillbirths plus mortality within the first week of life per 1000 births

^g^Extended perinatal mortality rate is stillbirths plus mortality within the first 28 days of life per 1000 births

Mothers residing in the mountains reported significantly higher PMR and EPMR than those who resided in the Terai or the hills (Table 2). Lower rates of perinatal and extended perinatal mortality were observed among women with aged between 25 to 49 years. Households with improved sanitation facilities, as well as those who used natural gas for cooking at home reported lower PMRs and EPMRs. Women who currently used contraceptives also reported lower PMR and EPMR.

**Table 2:** Characteristics of study population as weighted counts, and Perinatal Mortality (PM) and Extended Perinatal Mortality (EPM) rates with 95% Confidence Interval (CI) in Nepal (2001–2016), (*N* = 23,335)

Explanatory Variables	N^a^	PM Rate (95% CI)	EPM Rate (95% CI)
Community level factor			
Types of residence			
Urban	4395	31 (28, 33)	34 (32, 37)
Rural	18,940	44 (42, 47)	52 (49, 55)
Ecological zone			
Terai	12,169	42 (40, 45)	49 (46, 52)
Hill	9319	38 (35, 40)	45 (42, 47)
Mountain	1847	56 (53, 59)	67 (63, 70)
Socio-economic factor			
Wealth index			
Rich	4946	26 (24, 28)	29 (27, 31)
Middle	9644	44 (41, 47)	51 (48, 54)
Poor	8745	48 (45, 51)	57 (54, 60)
Religion			
Buddhist	1601	31 (28, 33)	41 (39, 44)
Hindu	19,639	42 (40, 45)	49 (46, 52)
Others^b^	2096	45 (42, 48)	48 (45, 51)
Ethnicity			
Brahmin/chettry	6323	37 (34, 39)	43 (40, 46)
Dalit	3716	51 (48, 54)	61 (58, 64)
Janajati	7647	37 (35, 40)	43 (40, 46)
Madhesi	5649	47 (44, 50)	55 (52, 58)
Mother education			
Secondary or higher	6151	29 (27, 32)	33 (31, 36)
Primary	4148	42 (39, 45)	48 (45, 51)
No education	13,036	47 (45, 50)	56 (53, 59)
Mother’s literacy level (*N* = 23,333)			
Can read part or whole of the sentence	11,391	32 (30, 35)	37 (34, 39)
Cannot read	11,942	51 (48, 54)	60 (57, 63)
Father education (*N* = 23,299)			
Secondary or higher	11,382	36 (33, 38)	41 (38, 44)
Primary	5923	43 (40, 46)	51 (48, 54)
No education	5994	52 (49, 55)	61 (58, 64)
Mother occupation (*N* = 23,333)			
Not working	5906	34 (32, 36)	39 (36, 41)
Agriculture	15,157	45 (43, 48)	53 (50, 56)
Non- Agriculture^c^	2270	36 (33, 38)	41 (38, 43)
Maternal factor			
Mother’s current age (years)			
25–49	14,131	39 (37, 42)	45 (42, 47)
15–18	821	67 (64, 70)	77 (73, 80)
19–24	8383	44 (41, 46)	53 (50, 56)
Maternal marital status			
Not currently married	219	32 (30, 34)	32 (30, 34)
Currently married	23,116	42 (39, 45)	49 (46, 52)
Birth order and birth interval			
2nd/3rd birth order, interval > 2 years	7065	19 (18, 21)	24 (22, 26)
1st birth order	7074	36 (33, 38)	44 (41, 46)
2nd/3rd birth order, interval ≤ 2 years	2613	42 (39, 45)	52 (49, 55)
4th or higher birth order, interval > 2 years	1727	82 (78, 85)	86 (83, 90)
4th or higher birth order, interval ≤ 2 years	4856	45 (42, 47)	59 (56, 62)
Environmental factor			
Types of drinking water source (*N* = 21,779)			
Piped water on premises	2990	31 (29, 33)	36 (34, 39)
Other improved drinking water sources	15,231	40 (38, 43)	47 (45, 50)
Unimproved drinking water sources	1434	45 (42, 47)	50 (47, 52)
Surface drinking water sources	2124	52 (49, 55)	59 (56, 62)
Types of sanitation facility (*N* = 21,775)			
Improved sanitation facilities	7931	30 (27, 32)	34 (31, 36)
Unimproved sanitation facilities	1924	42 (39, 45)	50 (47, 53)
Open defecation	11,920	47 (45, 50)	56 (53, 59)
Types of cooking fuel (*N* = 21,782)			
Natural gas	3035	22 (20, 24)	25 (23, 27)
Biomass energy	18,747	44 (41, 46)	51 (48, 54)
Health service factor			
Number of antenatal care (ANC) visits (*N* = 23,164)			
4+	8442	29 (27, 32)	34 (32, 37)
(1–3)	8330	37 (34, 39)	44 (41, 46)
No ANC	6392	40 (37, 42)	49 (47, 52)
Number of TT during pregnancy (*N* = 23,164)			
2 + TT	13,461	33 (31, 36)	39 (37, 42)
1 TT	3551	34 (32, 36)	40 (38, 43)
Never	6152	39 (36, 41)	48 (46, 51)
IFA supplementation during pregnancy(*N* = 23,168)			
Yes	13,460	33 (30, 35)	38 (35, 40)
No	9708	38 (36, 41)	47 (44, 50)
Place and assistance during delivery (*N* = 23,154)			
Health facility	6816	31 (29, 34)	35 (33, 37)
Home delivery with skilled attendants^d^	167	48 (45, 51)	54 (51, 57)
Home delivery without skilled attendants	16,171	36 (34, 39)	44 (42, 47)
Use of contraceptives			
Yes	8483	24 (22, 26)	29 (27, 32)
No	14,852	52 (49, 55)	60 (57, 63)

^a^Weighted study population. N varies between categories because of missing values

^b^other religion includes mainly Christians, Muslims, and Kirats

^c^Non-agriculture occupation includes skilled or professional jobs; ^d^Skilled attendants: doctors/nurses/midwives

### Multivariate analysis

Table 3 shows unadjusted and adjusted Odd Ratios (OR) for the association between perinatal mortality and exploratory variables, while Table 4 summarizes the corresponding ORs for extended perinatal mortality.

**Table 3:** Unadjusted and adjusted Odd Ratios (OR) for factors associated with perinatal mortality in Nepal, 2001–2016 (*N* = 23,335)

Explanatory Variables	Unadjusted	Adjusted
	OR (95% CI)	OR (95% CI)
Year of survey		
2001	1.00 (Reference)	1.00 (Reference)
2006	0.92 (0.78, 1.10)	0.95 (0.78, 1.16)
2011	0.77 (0.64, 0.93)	0.98 (0.80, 1.21)
2016	0.62 (0.51, 0.77)	0.72 (0.56, 0.92)
Community level factor		
Types of residence		
Urban	1.00 (Reference)	
Rural	1.46 (1.20, 1.79)	
Ecological zone		
Terai	1.00 (Reference)	1.00 (Reference)
Hill	0.89 (0.75, 1.05)	1.02 (0.83, 1.26)
Mountain	1.35 (1.09, 1.66)	1.44 (1.07, 1.95)
Socio-economic factor		
Wealth index		
Rich	1.00 (Reference)	
Middle	1.73 (1.41, 2.13)	
Poor	1.93 (1.57, 2.39)	
Religion		
Buddhist	1.00 (Reference)	
Hindu	1.37 (0.99, 1.91)	
Others including Muslim and Christian	1.47 (0.97, 2.21)	
Ethnicity		
Brahmin/chettri	1.00 (Reference)	1.00 (Reference)
Dalit	1.44 (1.13, 1.82)	1.30 (1.02, 1.65)
Janajati	1.02 (0.83, 1.26)	1.05 (0.84, 1.31)
Madhesi	1.31 (1.07, 1.62)	1.28 (0.99, 1.65)
Mother education		
Secondary or higher	1.00 (Reference)	
Primary	1.44 (1.11, 1.85)	
No education	1.64 (1.33, 2.03)	
Mother’s literacy level		
Can read part or whole of the sentence	1.00 (Reference)	
Cannot read	1.42 (1.24, 1.63)	
Father education		
Secondary or higher	1.00 (Reference)	
Primary	1.21 (1.00, 1.46)	
No education	1.47 (1.23, 1.77)	
Mother occupation		
Not working	1.00 (Reference)	
Agriculture	1.28 (1.05, 1.56)	
Non-agriculture	0.95 (0.66, 1.36)	
Maternal factor		
Mother’s current age (years)		
25–49	1.00 (Reference)	1.00 (Reference)
15–18	1.76 (1.27, 2.45)	1.99 (1.30, 3.09)
19–24	1.12 (0.95, 1.33)	1.71 (1.39, 2.11)
Maternal marital status		
Not currently married	1.00 (Reference)	
Currently married	1.43 (0.63, 3.26)	
Birth order and birth interval		
2nd/3rd birth order, interval > 2 years	1.00 (Reference)	1.00 (Reference)
1st birth order	1.90 (1.54, 2.35)	1.32 (1.03, 1.69)
2nd/3rd birth order, interval ≤ 2 years	2.20 (1.71, 2.85)	1.83 (1.39, 2.42)
4th or higher birth order, interval > 2 years	4.48 (3.67, 5.47)	3.27 (2.58, 4.15)
4th or higher birth order, interval ≤ 2 years	2.35 (1.76, 3.12)	2.41 (1.76, 3.29)
Environmental factor		
Types of drinking water source		
Piped water on premises	1.00 (Reference)	
Other improved drinking water sources	1.30 (1.00, 1.68)	
Unimproved drinking water sources	1.45 (1.00, 2.12)	
Surface drinking water sources	1.70 (1.24, 2.35)	
Types of sanitation facility		
Improved sanitation facilities	1.00 (Reference)	
Unimproved sanitation facilities	1.44 (1.06, 1.95)	
Open defecation	1.63 (1.37, 1.94)	
Types of cooking fuel		
Natural gas	1.00 (Reference)	1.00 (Reference)
Biomass energy	2.05 (1.54, 2.73)	1.46 (1.08, 1.97)
Health service factor		
Number of antenatal care (ANC) visits		
4+	1.00 (Reference)	
(1–3)	1.25 (1.04, 1.51)	
No ANC	1.37 (1.10, 1.70)	
Number of TT during pregnancy		
2 + TT	1.00 (Reference)	
1 TT	1.02 (0.80, 1.30)	
Never	1.18 (0.97, 1.43)	
IFA supplementation during pregnancy		
Yes	1.00 (Reference)	
No	1.18 (0.99, 1.41)	
Place and assistance during delivery		
Health facility	1.00 (Reference)	
Home delivery with skilled attendants	1.59 (0.76, 3.32)	
Home delivery without skilled attendants	1.12 (0.94, 1.32)	
Use of contraceptives		
Yes	1.00 (Reference)	1.00 (Reference)
No	2.25 (1.87, 2.70)	1.93 (1.61, 2.31)

**Table 4:** Unadjusted and adjusted Odd Ratios (OR) for factors associated with extended perinatal mortality in Nepal, 2001–2016 (*N* = 23,335)

Explanatory Variables	Unadjusted	Adjusted
	OR (95% CI)	OR (95% CI)
Year of survey		
2001	1.00 (Reference)	1.00 (Reference)
2006	0.92 (0.79, 1.08)	0.98 (0.82, 1.18)
2011	0.70 (0.58, 0.83)	0.88 (0.73, 1.08)
2016	0.58 (0.48, 0.70)	0.69 (0.54, 0.86)
Types of residence		
Urban	1.00 (Reference)	
Rural	1.54 (1.27, 1.86)	
Ecological zone		
Terai	1.00 (Reference)	1.00 (Reference)
Hill	0.91 (0.78, 1.06)	0.99 (0.84, 1.18)
Mountain	1.39 (1.15, 1.68)	1.37 (1.06, 1.76)
Socio-economic factor		
Wealth index		
Rich	1.00 (Reference)	
Middle	1.66 (1.33, 2.08)	
Poor	1.53 (1.13, 1.81)	
Religion		
Buddhist	1.00 (Reference)	
Hindu	1.21 (0.89, 1.63)	
Others including Muslim and Christian	1.18 (0.80, 1.74)	
Ethnicity		
Brahmin/chettri	1.00 (Reference)	
Dalit	1.45 (1.16, 1.80)	
Janajati	1.00 (0.83, 1.22)	
Madhesi	1.28 (1.05, 1.57)	
Mother education		
Secondary or higher	1.00 (Reference)	
Primary	1.46 (1.14, 1.86)	
No education	1.73 (1.42, 2.11)	
Mother’s literacy level		
Can read part or whole of the sentence	1.00 (Reference)	1.00 (Reference)
Cannot read	1.51 (1.33, 1.72)	1.23 (1.05, 1.45)
Father education		
Secondary or higher	1.00 (Reference)	
Primary	1.27 (1.08, 1.50)	
No education	1.52 (1.28, 1.81)	
Mother occupation		
Not working	1.00 (Reference)	
Agriculture	1.32 (1.09, 1.59)	
Non-agriculture	0.94 (0.68, 1.32)	
Maternal factor		
Mother’s current age (years)		
25–49	1.00 (Reference)	1.00 (Reference)
15–18	1.79 (1.32, 2.42)	2.15 (1.45, 3.18)
19–24	1.19 (1.03, 1.39)	1.78 (1.47, 2.15)
Maternal marital status		
Not currently married	1.00 (Reference)	
Currently married	1.68 (0.74, 3.82)	
Birth order and birth interval		
2nd/3rd birth order, interval > 2 years	1.00 (Reference)	1.00 (Reference)
1st birth order	1.86 (1.54, 2.25)	1.30 (1.04, 1.63)
2nd/3rd birth order, interval ≤ 2 years	2.17 (1.72, 2.74)	1.80 (1.41, 2.31)
4th or higher birth order, interval > 2 years	3.79 (3.15, 4.55)	2.75 (2.20, 3.43)
4th or higher birth order, interval ≤ 2 years	2.52 (1.96, 3.25)	2.56 (1.94, 3.38)
Environmental factor		
Types of drinking water source		
Piped water on premises	1.00 (Reference)	
Other improved drinking water sources	1.33 (1.05, 1.69)	
Unimproved drinking water sources	1.40 (0.97, 2.00)	
Surface drinking water sources	1.68 (1.25, 2.26)	
Types of sanitation facility		
Improved sanitation facilities	1.00 (Reference)	
Unimproved sanitation facilities	1.51 (1.10, 2.07)	
Open defecation	1.70 (1.44, 2.01)	
Types of cooking fuel		
Natural gas	1.00 (Reference)	1.00 (Reference)
Biomass energy	2.11 (1.60, 2.79)	1.44 (1.09, 1.91)
Health service factor		
Number of antenatal care (ANC) visits		
4+	1.00 (Reference)	
(1–3)	1.29 (1.07, 1.54)	
No ANC	1.47 (1.21, 1.80)	
Number of TT during pregnancy		
2 + TT	1.00 (Reference)	
1 TT	1.03 (0.83, 1.29)	
Never	1.25 (1.04, 1.50)	
IFA supplementation during pregnancy		
Yes	1.00 (Reference)	
No	1.25 (1.07, 1.47)	
Place and assistance during delivery		
Health facility	1.00 (Reference)	
Home delivery with skilled attendants	1.69 (0.87, 3.31)	
Home delivery without skilled attendants	1.23 (1.05, 1.43)	
Use of contraceptives		
Yes	1.00 (Reference)	1.00 (Reference)
No	2.10 (1.77, 2.51)	1.79 (1.52, 2.11)

### Factors associated with perinatal mortality (PM)

After adjusting for potential explanatory variables, perinatal mortality has decreased significantly by 28% (aOR: 0.72; 95% CI: 0.56, 0.92) between 2001 and 2016. Women residing in the mountain ecological zone and women of the dalit ethnic group had increased risk of perinatal mortality compared to those who reside in the terai ecological zone and belong to Brahmin/Chettri ethnic group. A significant increment of perinatal mortality was observed among women having 4th or higher birth order with any years of interval compared to women having 2nd or 3rd birth order with > 2 years of interval. Women aged 15 to 18 years or19 to 24 years, and those not using contraception reported higher risk of perinatal mortality compared to those with aged 25 to 49 years, and who were using contraception. Women who used biomass energy for cooking at home had significantly higher odds of having perinatal mortality compared to those who used natural gas for cooking.

In the final model of PM, when types of cooking fuel were removed and replaced by household wealth index, we found that women from the middle households were more likely to report PM (aOR: 1.44; 95% CI: 1.13, 1.83) compared to their richer counterparts.

### Factors associated with extended perinatal mortality (EPM)

Our results showed similar factors to be associated with EPM as those mentioned for PM. We replaced household wealth index with types of cooking fuel in the final model; and our results indicated that women from middle households were more likely to report EPM (aOR: 1.42; 95% CI: 1.13, 1.78) compared to their richer counterparts.

## Discussion

This study identified more distant factors associated with PM and EPM in Nepal. Over the study period (2001–2016), there has been a significant decline in PM and EPM. However, the pace of progress is not sufficient enough to achieve SDG target of 12 or fewer perinatal deaths per 1000 births by the year 2030. We found that the factors that were consistent across PM and EPM were ecological zone, household wealth index, birth order and birth interval; maternal age, use of contraceptives, and type of cooking fuel. Similarly, the study also found that maternal ethnic background was associated with PM; whereas maternal literacy level was found to be associated with EPM.

In this study, the odds of PM and EPM were significantly higher among women residing in the mountain ecological zone compared to those who lived in the terai ecological zone. This finding is consistent with a past study conducted in a landlocked country of Peru which has shown that high altitude (≥3000 m above the sea level) was associated with perinatal mortality [26]. Similarly, a study conducted in Nepal revealed that mothers who lived in mountainous region, with altitude ranges from 4877 m to 8848 m above the sea level were significantly more likely to report an increased risk of perinatal mortality [27]. Aadditionally, higher perinatal mortality in the mountains could be connected to access to services. Absence of adequate medical facilities would be an important contributor to the increased risk of perinatal mortality due to difficulties in timely accessing maternal and newborn health care services including emergency obstetric care during labour.

Women who were illiterate had a higher risk of EPM. This finding was consistent with a prospective longitudinal study conducted in Northwest Ethiopia [19]. Similarly, a case-control study conducted in Mashonaland East Province of Zimbabwe revealed that mothers who completed Primary education or no schooling were 5.4 times more likely to report PM compared with educated mothers [28]. These findings contradict a study carried out in Bangladesh that used prospective data on maternal morbidity which indicated that women who completed secondary or more level of education reported higher risks of perinatal mortality compared with women with no schooling [29].

Higher PM and EPM among younger age women reported in this study were similar to those reported in a population-based nested case-control conducted in Ethiopia [30]. Younger women are more likely to receive inadequate health care including family planning than older women due to the low level of women’s autonomy regarding health care decisions in the households [31], psychological problems and less social support from family members [32]. Higher odds of perinatal mortality among younger women may be due to inadequate weight gain during pregnancy, low socioeconomic status, and inadequate prenatal care [33]. In Nepal, early marriage and pregnancy is still a common practice with about 17% of girls married before the age of 19 years [15]. Early marriage is a deeply rooted and widely practised socio-cultural norm. For example, in the regional part of Nepal, bride family are required to pay extra money when young unmarried girls grow older [34].

In agreement with our findings a previous study in Nepal and India showed that mothers with 4th or higher birth order had higher probability of perinatal death [9, 35]. Obstetric complications are important causes of perinatal mortality in South Asia [17]; and a previous study has suggested that women with 4th or higher birth order were at greater risk of having any obstetric complication compared to women with birth order < 3 [36].

In Asia, while environmental factors (unimproved water, sanitation, and smoke from unimproved cooking fuel) are considered among the ten leading risks for disease [37], Nepal has made substantial improvements in the use of improved drinking water sources and sanitation facilities over the past 15 years [12–15]. Yet, the majority (over 65%) of households in the same period have constantly been using biomass energy for cooking at home [12–15]. The finding of an increased risk of PM and EPM has also been reported in a hospital-based surveillance and case-control study, linked with a population survey in India [38]. Past studies suggested that chronic exposure to carbon monoxide from biomass energy will deteriorate newborn respiratory health that may lead to a lower chance of survival especially in the first month of infant life [39]. In Nepal, natural gas is expensive; and the use of natural gas is more prevalent among rich households [12–15]. Our study also revealed that women from a rich household wealth index were less likely to report PM and EMP compared to women from a lower household wealth index. Access to affordable renewal energy both at home and community could benefit health and thus help in reducing perinatal deaths.

A plausible reason for the finding of a higher PM among Dalit women may be because Dalits were ethnic minority women, less empowered, and socio-economically marginalised with limited ability to improve their health and the health of their newborn. It has been documented that 90% of total Dalit populations living below the poverty line in Nepal have limited access to health care [40].

The higher perinatal mortality rate in home births with skilled birth attendants compared to those without skilled birth attendants in this study may be the reflection of high-risk pregnancies which may be difficult to manage in home settings.

This study has many strengths, including the use of the pooled Nepal DHS (2001–2016) with large sample sizes, and higher response rates (almost 97%). Second, the study investigated factors associated with PM and EPM using household national-wide representative surveys; and findings from a population-based study can be used by public health practitioners to formulate and inform national level policies to improve fetal and newborn survival. Third, the same questionnaires were used to collect information in all four surveys which increase accuracy and promote coherence of the data used for analysis. Despite these strengths, this study also has limitations. First, information about duration of pregnancy and birth intervals (which may be complex for illiterate women) may lead to reporting bias. Second, twin stillbirths are recorded as one stillbirth in the calendar that may undercount the number of total stillbirths, but they are very rare events. Third, this study is limited by the fact that data on important explanatory variables such as a history of previous stillbirth, prematurity, fetal growth, low birthweight, maternal anaemia, obstetric complications such as breech delivery, and birth asphyxia were not collected in the four NDHS. Fourthly, maternal body mass index, an important confounder, was not included in our multivariate analysis due to large numbers of missing data (23%) which if included could have biased the results.

## Conclusions

Our analyses examined more distant factors associated with PM and EPM in Nepal using pooled population-based surveys for the year 2001, 2006, 2011, and 2016. Women who lived in the mountains, women who did not use contraceptives at the time of the survey, younger women, and women having 4th or higher birth order had significantly higher risks of PM and EPM. At household level, educating illiterate women about the benefits of using contraceptives is required to further reduce perinatal mortality. At community level, modern energy technologies such as the use of liquefied petroleum gas or electricity to all households may help to reduce perinatal mortality; and these interventions should target rural women and women from low socioeconomic households.

## Additional files

Additional file 1: Adjusted Odd Ratios (aOR) for factors associated with perinatal mortality in Nepal, 2001–2016 (*N* = 23,335). (DOCX 19 kb)

Additional file 2: Adjusted Odd Ratios (aOR) for factors associated with extended perinatal mortality in Nepal, 2001–2016 (*N* = 23,335). (DOCX 19 kb)

## Abbreviations

ANC: Antenatal Care
EPM: Extended Perinatal Mortality
EPMR: Extended Perinatal Mortality Rate
IFA: Iron and Folic Acid
NDHS: Nepal Demographic and Health Survey
OR: Odd Ratio
PM: Perinatal Mortality
PMR: Perinatal Mortality Rate
TT: Tetanus Toxoid
