# Supplementary material for: Factors associated with perinatal mortality in Nepal: evidence from Nepal demographic and health survey 2001–2016
Source: BMC Pregnancy Childbirth. 2019 Mar 11;19:88. doi: 10.1186/s12884-019-2234-6 (PMC6417106; doi:10.1186/s12884-019-2234-6)
Supplement: Supplementary file 1 — Adjusted Odd Ratios (aOR) for factors associated with perinatal mortality in Nepal, 2001–2016 (N = 23,335). (DOCX 19 kb) [file 12884_2019_2234_MOESM1_ESM.docx]

**Additional file 1**. Adjusted Odd Ratios (aOR) for factors associated with perinatal mortality in Nepal, 2001-2016 (N=23,335)

| Exploratory Variables | Results using each year of survey weights | | Results using re-normalization of survey weights add up to 1 | |
| --- | --- | --- | --- | --- |
|  | aOR (95% CI) | P-Value | aOR (95% CI) | P-Value |
| Year of survey |  |  |  |  |
| 2001 | 1.00(Reference) |  | 1.00(Reference) |  |
| 2006 | 0.95(0.78, 1.16) | 0.637 | 0.97(0.78, 1.20) | 0.778 |
| 2011 | 0.98(0.80, 1.21) | 0.856 | 0.98(0.77, 1.25) | 0.897 |
| 2016 | 0.72(0.56, 0.92) | 0.008 | 0.73(0.55, 0.96) | 0.024 |
| *Community level factor* |  |  |  |  |
| Ecological zone |  |  |  |  |
| Terai | 1.00(Reference) |  | 1.00(Reference) |  |
| Hill | 1.02(0.83, 1.26) | 0.825 | 1.02(0.83, 1.25) | 0.871 |
| Mountain | 1.44(1.07, 1.95) | 0.016 | 1.45(1.15, 1.82) | 0.002 |
| *Socio-economic factor* |  |  |  |  |
| Ethnicity |  |  |  |  |
| Brahmin/chettri | 1.00(Reference) |  | 1.00(Reference) |  |
| Dalit | 1.30(1.02, 1.65) | 0.032 | 1.29(1.00, 1.65) | 0.045 |
| Janajati | 1.05(0.84, 1.31) | 0.655 | 1.03(0.83, 1.28) | 0.765 |
| Madhesi | 1.28(0.99, 1.65) | 0.062 | 1.26(0.96, 1.64) | 0.088 |
| *Maternal factor* |  |  |  |  |
| Mother's current age (years) |  |  |  |  |
| 25-49 | 1.00(Reference) |  | 1.00(Reference) |  |
| 15-18 | 1.99(1.30, 3.09) | 0.002 | 1.96(1.20, 3.22) | 0.007 |
| 19-24 | 1.71(1.39, 2.11) | <0.001 | 1.69(1.24, 2.29) | 0.001 |
| Birth order and birth interval |  |  |  |  |
| 2nd/3rd birth order, interval >2 years | 1.00(Reference) |  | 1.00(Reference) |  |
| 1^st^ birth order | 1.32(1.03, 1.69) | 0.029 | 1.32(1.00, 1.73) | 0.049 |
| 2^nd^/3^rd^ birth order, interval ≤2 years | 1.83(1.39, 2.42) | <0.001 | 1.86(1.35, 2.55) | <0.001 |
| 4^th^ or higher birth order, interval >2 years | 3.27(2.58, 4.15) | <0.001 | 3.23(2.38, 4.40) | <0.001 |
| 4^th^ or higher birth order, interval ≤2 years | 2.41(1.76, 3.29) | <0.001 | 2.38(1.66, 3.42) | <0.001 |
| *Environmental factor* |  |  |  |  |
| Types of cooking fuel |  |  |  |  |
| Natural gas | 1.00(Reference) |  | 1.00(Reference) |  |
| Biomass energy | 1.46(1.08, 1.97) | 0.015 | 1.43(1.04, 1.97) | 0.026 |
| *Health service factor* |  |  |  |  |
| Use of contraceptives |  |  |  |  |
| Yes | 1.00(Reference) |  | 1.00(Reference) |  |
| No | 1.93(1.61, 2.31) | <0.001 | 1.93(1.58, 2.36) | <0.001 |
