# Supplementary material for: Factors associated with perinatal mortality in Nepal: evidence from Nepal demographic and health survey 2001–2016
Source: BMC Pregnancy Childbirth. 2019 Mar 11;19:88. doi: 10.1186/s12884-019-2234-6 (PMC6417106; doi:10.1186/s12884-019-2234-6)
Supplement: Supplementary file 2 — Adjusted Odd Ratios (aOR) for factors associated with extended perinatal mortality in Nepal, 2001–2016 (N = 23,335). (DOCX 19 kb) [file 12884_2019_2234_MOESM2_ESM.docx]

| Exploratory Variables | Results using each year of survey weights | | Results using re-normalization of survey weights add up to 1 | |
| --- | --- | --- | --- | --- |
|  | aOR (95% CI) | P-Value | aOR (95% CI) | P-Value |
| Year of survey |  |  |  |  |
| 2001 | 1.00(Reference) |  | 1.00(Reference) |  |
| 2006 | 0.98(0.82, 1.18) | 0.861 | 0.99(0.80, 1.22) | 0.946 |
| 2011 | 0.88(0.73, 1.08) | 0.219 | 0.89(0.71, 1.12) | 0.326 |
| 2016 | 0.69(0.54, 0.86) | 0.001 | 0.69(0.54, 0.89) | 0.005 |
| *Community level factor* |  |  |  |  |
| Ecological zone |  |  |  |  |
| Terai | 1.00(Reference) |  | 1.00(Reference) |  |
| Hill | 0.99(0.84, 1.18) | 0.928 | 0.99(0.83, 1.18) | 0.911 |
| Mountain | 1.37(1.06, 1.76) | 0.015 | 1.36(1.13, 1.65) | 0.001 |
| *Socio-economic factor* |  |  |  |  |
| Mother's literacy level |  |  |  |  |
| Can read part or whole of the sentence | 1.00(Reference) |  | 1.00(Reference) |  |
| Cannot read | 1.23(1.05, 1.45) | 0.012 | 1.24(1.04, 1.49) | 0.018 |
| *Maternal factor* |  |  |  |  |
| Mother's current age (years) |  |  |  |  |
| 25-49 | 1.00(Reference) |  | 1.00(Reference) |  |
| 15-18 | 2.15(1.45, 3.18) | <0.001 | 2.14(1.39, 3.31) | 0.001 |
| 19-24 | 1.78(1.47, 2.15) | <0.001 | 1.76(1.35, 2.29) | <0.001 |
| Birth order and birth interval |  |  |  |  |
| 2nd/3rd birth order, interval >2 years | 1.00(Reference) |  | 1.00(Reference) |  |
| 1^st^ birth order | 1.30(1.04, 1.63) | 0.021 | 1.30(1.02, 1.67) | 0.037 |
| 2^nd^/3^rd^ birth order, interval ≤2 years | 1.80(1.41, 2.31) | <0.001 | 1.82(1.39, 2.39) | <0.001 |
| 4^th^ or higher birth order, interval >2 years | 2.75(2.20, 3.43) | <0.001 | 2.73(2.08, 3.57) | <0.001 |
| 4^th^ or higher birth order, interval ≤2 years | 2.56(1.94, 3.38) | <0.001 | 2.54(1.84, 3.51) | <0.001 |
| *Environmental factor* |  |  |  |  |
| Types of cooking fuel |  |  |  |  |
| Natural gas | 1.00(Reference) |  | 1.00(Reference) |  |
| Biomass energy | 1.44(1.09, 1.91) | 0.011 | 1.42(1.06, 1.92) | 0.020 |
| *Health service factor* |  |  |  |  |
| Use of contraceptives |  |  |  |  |
| Yes | 1.00(Reference) |  | 1.00(Reference) |  |
| No | 1.79(1.52, 2.11) | <0.001 | 1.80(1.50, 2.16) | <0.001 |

**Additional file 2**. Adjusted Odd Ratios (aOR) for factors associated with extended perinatal mortality in Nepal, 2001-2016 (N=23,335)
